# Supplementary material for: A Novel GUCA1A Variant Associated with Cone Dystrophy Alters cGMP Signaling in Photoreceptors by Strongly Interacting with and Hyperactivating Retinal Guanylate Cyclase
Source: Int J Mol Sci. 2021 Oct 6;22(19):10809. doi: 10.3390/ijms221910809 (PMC8509414; doi:10.3390/ijms221910809)
Supplement: Supplementary file 1 [file ijms-22-10809-s001.zip › ijms-1392057-supplementary.pdf]

International Journal of
Molecular Sciences

MDPI

Supplementary Materials

A Novel *GUCA1A* Variant Associated with Cone Dystrophy Alters cGMP Signaling in Photoreceptors by Strongly Interacting with and Hyperactivating Retinal Guanylate Cyclase

Amedeo Biasi, Valerio Marino, Giuditta Dal Cortivo, Paolo Enrico Maltese, Antonio Mattia Modarelli, Matteo Bertelli, Leonardo Colombo and Daniele Dell'Orco

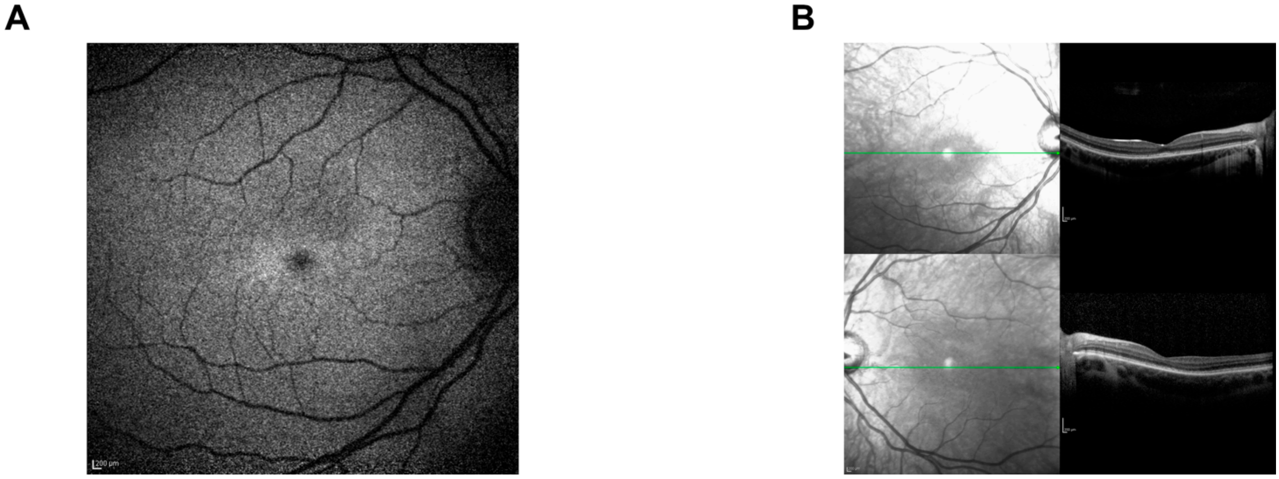

Figure S1. (A) Fundus image of the right eye and (B) SD-OCT scans of right and left eye of patient II:2 at the age of 10. Line corresponding to interdigitation zone is shown in green.

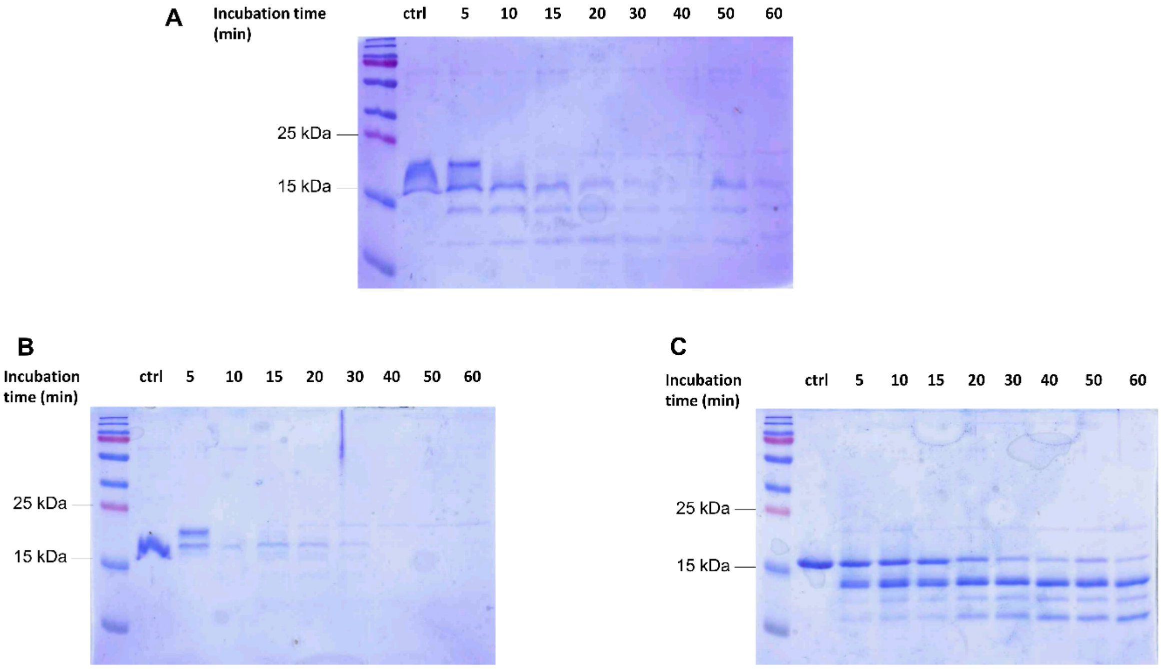

Figure S2. Time-dependent limited proteolysis of 20 µM GCAP1 WT in the presence of (A) 2 mM EDTA (ctrl) or 2 mM EDTA + 0.3 µM trypsin, (B) 1 mM EGTA + 1.1 mM Mg^{2+} (ctrl) or 1 mM EGTA + 1.1 mM Mg^{2+} + 0.3 µM trypsin, (C) 1 mM

Mg^{2+} and 1 mM Ca^{2+} (ctrl) or 1 mM Mg^{2+} and 1 mM Ca^{2+} + 0.3 μM trypsin. Incubation times were (from left to right): 5-10-15-20-30-40 and 60 minutes for each condition.

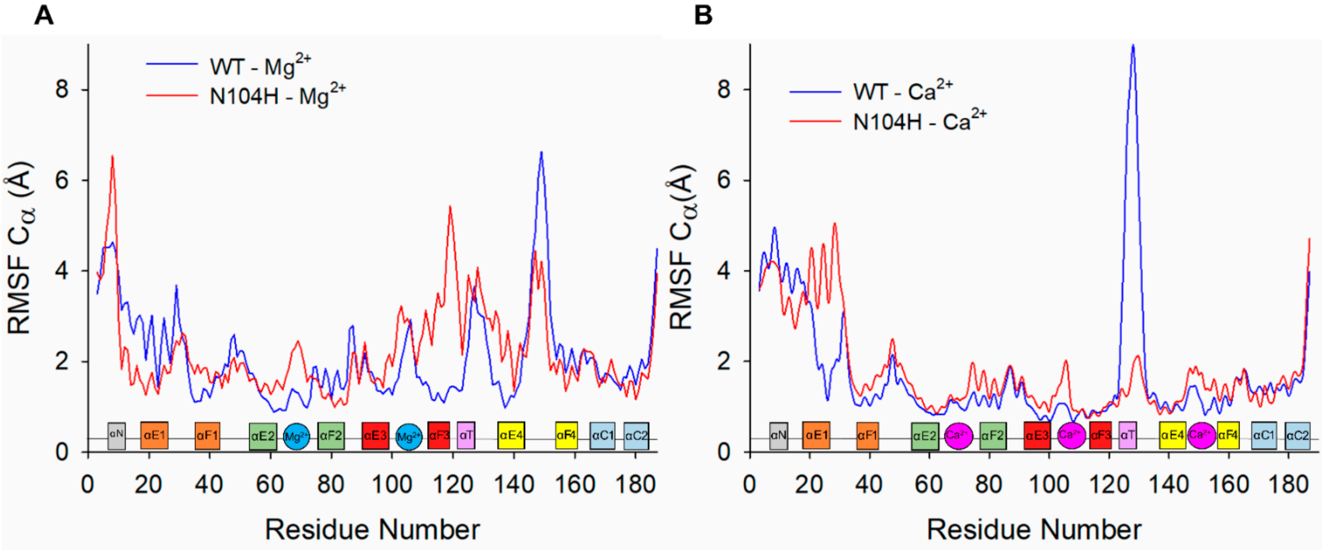

Figure S3. Cα-RMSF profiles (calculated over 2 μs MD simulations) of (A) Mg^{2+}-bound and (B) Ca^{2+}-loaded WT (blue line) and N104H (red line) GCAP1. Insets show secondary structure elements represented with the same coloring scheme as Fig. 5. Ca^{2+} and Mg^{2+} ions are represented respectively as pink and blue circles.

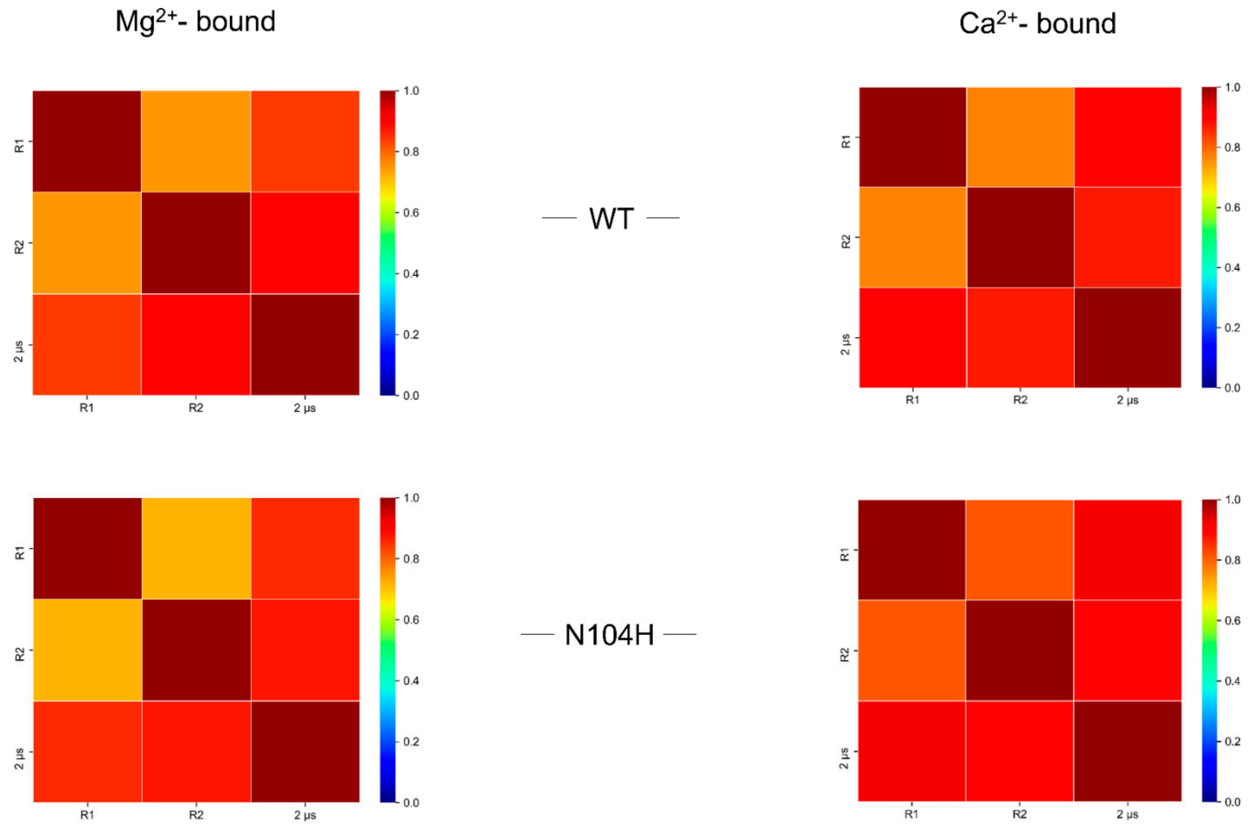

Figure S4. RMSIP of the first 20 principal components of the two 1 μs MD simulation replicas (R1 and R2) and of the concatenated trajectories of WT- and N104H-GCAP1 in their Mg^{2+}-bound and Ca^{2+}-loaded forms.
